# Composition and Function of Gut Microbiome: From Basic Omics to Precision Medicine

**DOI:** 10.3390/genes17010116

**Published:** 2026-01-22

**Authors:** Yan Ma, Lamei Wang, Haitao Hu, Audrey Ruei-En Shieh, Edward Li, Dongdong He, Lin He, Zhong Liu, Thant Mon Paing, Xinhua Chen, Yangchun Cao

**Affiliations:** 1 College of Animal Science and Technology, Northwest A&F University, Yangling 712100, China; 2 Division of Gastroenterology, Department of Medicine, Beth Israel Deaconess Medical Center, Harvard Medical School, Boston, MA 02115, USA; audrey.shieh@gmail.com (A.R.-E.S.); seanliuharvard@gmail.com (Z.L.); 3 University of British Columbia, Vancouver, BC V6T 1Z2, Canada; helin0429andrew@gmail.com; 4 Department of Animal Science, Yezin Agricultural University, Nay Pyi Taw 15013, Myanmar

**Keywords:** human microbiome, health and disease, host–microbe interactions, metagenomics, multi-omics approaches

## Abstract

The gut microbiome is defined as the collective assembly of microbial communities inhabiting the gut, along with their genes and metabolic products. The gut microbiome systematically regulates host metabolism, immunity, and neuroendocrine homeostasis via interspecies interaction networks and inter-organ axes. Given the importance of the gut microbiome to the host, this review integrates the composition, function, and genetic basis of the gut microbiome with host genomics to provide a systematic overview of recent advances in microbiome–host interactions. This encompasses a complete technological pipeline spanning from in vitro to in vivo models to translational medicine. This technological pipeline spans from single-bacterium CRISPR editing, organoid–microbiome co-culture, and sterile/humanized animal models to multi-omics integrated algorithms, machine learning causal inference, and individualized probiotic design. It aims to transform microbiome associations into precision intervention strategies that can be targeted and predicted for clinical application through interdisciplinary research, thereby providing the cornerstone of a new generation of precision treatment strategies for cancer, metabolic, and neurodegenerative diseases.

## 1. Introduction

The human body is not only composed of human cells but is also a superorganism that coexists with trillions of microorganisms. The collective assemblage of all microorganisms (including bacteria, archaea, fungi, viruses, and protozoa) that inhabit the internal and external surfaces of the human body, along with their genetic information, metabolic by-products, and the surrounding environment, is collectively referred to as the human microbiome [1]. The gut microbiome is the most extensively studied and influential part among them and is regarded as the “second genome” and “living metabolic organ” of the human body [2]. Since the European Union launched the “Metagenomics of the Human Intestinal Tract (MetaHIT)” in 2008, a large number of studies have systematically constructed disease–microbiome–metabolism association maps using methods such as microbial community structure profiling and reference gene catalogs [3].

The gut microbiome is not a fixed organ present at birth but rather is a dynamic ecosystem that evolves throughout the host’s life. In the early stage of life, the microbiome is primarily associated with the mode of birth delivery and feeding practices. Subsequently, it gradually colonizes and begins to proliferate within the gut [4,5,6,7]. The diversity and abundance of the gut microbiome gradually increase until the age of 3 years. As individuals grow, the composition of the gut microbiome matures and stabilizes, yet it remains subject to the influence of factors such as diet, lifestyle, medication use, and environmental changes [8,9,10]. Despite individual variations, a healthy microbiome typically exhibits high species diversity and functional redundancy. This stability is essential for maintaining the host’s physiological balance.

**Figure 1:**
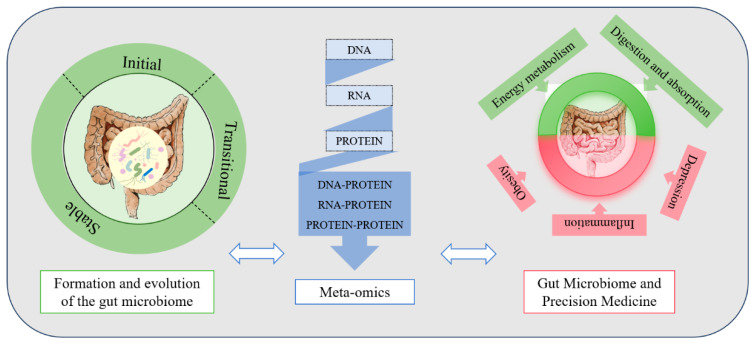
Gut microbiome succession influencing human health and disease.

In recent years, research on the gut microbiome has emerged as a major focus in the life sciences and medicine (Figure 1). Its significance is reflected in the following aspects:

- The Link to Health and Disease: The gut microbiome plays a dual role, participating in essential host functions such as nutrient metabolism, immune regulation, and neuro-signaling while also being intimately linked to the pathogenesis of a wide range of conditions [11,12,13,14,15], including obesity, type 2 diabetes mellitus (T2DM), inflammatory bowel disease (IBD), autoimmune diseases, and certain neuropsychiatric disorders (such as depression and Alzheimer’s disease) [16,17,18,19].
- Technology-Driven Research Revolution: The rapid advancement of techniques, including 16S rRNA sequencing, metagenomics, metatranscriptomics, and multi-omics, has revolutionized our understanding of the gut microbiome. It allows us to conduct in-depth research into the complexity of the gut microbiome from multiple perspectives, including its composition, function, and dynamic changes [20,21,22,23].
- Research Value of Host–Microbe Interactions: The gut microbiome engages in dynamic interactions with the host’s genes and immune system through its metabolic products (such as SCFAs and toxins) [24,25,26]. This interactive network offers new insights into the molecular mechanisms underlying disease and may serve as a potential therapeutic target for treating diseases.

This review aims to examine the establishment and core physiological functions of the gut microbiome and summarize its role in health and disease, thereby advancing the understanding of microbiome-mediated mechanisms and their impact on health and disease management. Integrating the existing literature with clinical research not only deepens our understanding of the core mechanisms by which the gut microbiome influences health maintenance and disease onset but also provides a scientific foundation for developing innovative therapeutic strategies targeting the gut microbiome.

## 2. Formation, Evolution, and Function of the Gut Microbiome

The health status of the gut microbiome can be described by global parameters (richness and diversity), compositional characteristics (phyla and taxa), and functional characteristics (metabolic patterns and pathways). The gut microbiome continuously changes as its host develops and grows. It is gradually formed from the moment an infant is born, as the infant transitions from breast milk to complementary foods, and eventually to a full diet, ultimately developing into a relatively stable, unique individual ecosystem [27,28,29,30]. The trillions of microorganisms and their metabolites in the human gut perform multiple physiological functions, including the digestion and metabolism of nutrients, participation in the host immune system, and resistance to pathogen invasion [31]. Based on the above key points, this review summarizes recent advances in gut microbiome research, focusing on initial colonization, stabilization, and physiological functions of the gut microbiome.

### 2.1. Initial Establishment

**Figure 2:**
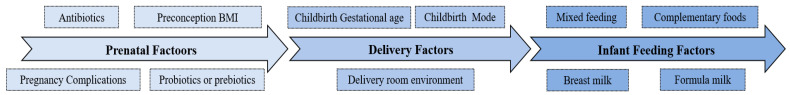
The early life stages of gut microbiota colonization and development.

The scientific community has long held that the newborn gut is sterile at birth, with initial colonization beginning during delivery through contact with the maternal birth canal, skin, and environmental microorganisms [5,32]. Recent studies using high-throughput sequencing technology have detected microorganisms in the placenta, amniotic fluid, and meconium, suggesting that low-level microbial exposure may occur in utero [33,34]. The initial establishment of the human gut microbiome occurs during infancy. The early life stages of gut microbiome colonization and development are shown in Figure 2 [35,36,37,38,39]. Thereafter, the microbiota undergoes highly dynamic changes throughout infancy and early childhood, ultimately achieving further development and stabilization during childhood.

At 0–1 months of age, the earliest colonizing aerobic and facultative anaerobic bacteria consume oxygen, creating an anaerobic environment conducive to subsequent anaerobic colonization. At this stage, the mode of delivery exerts a significant influence: infants delivered vaginally exhibit a higher relative abundance of Bacteroides, while those delivered via cesarean section show higher relative abundance of Klebsiella, Haemophilus, and Veillonella in their intestines. At 1–6 months of age, strict anaerobes begin to proliferate extensively, yet diversity remains consistently low. Feeding practices exert a pronounced influence during this phase, with *Bifidobacterium* dominating the gut microbiome of breastfed infants [40]. At 6–18 months of age, the relative abundance of the genus *Bacteroides* increases. The impact of diet during this stage is particularly pronounced. With the introduction of solid complementary foods, gut microbiota species richness increases significantly, accompanied by a rise in α diversity [41]. At 18–36 months of age, the gut microbiome begins to stabilize as it continues to adapt to environmental factors. Despite being dominated by the phyla Firmicutes and Bacteroidetes, the diversity and evenness of the microbiome contained were at a low level. At 0–36 months of age, gut microbiota constantly evolves with age. Studies show that the gut microbiome of 3-year-olds becomes relatively stable and similar to an adult’s [42]. Even by age 5 in the preschool stage, the complexity of the gut microbiota still falls short of that seen in adults [43,44]. The succession process of the gut microbiome community starts to establish communities with birth and after reaching the peak of adolescence. Then the similarity of intestinal microflora tends to be similar to that of adults, but there are still some differences due to the environment and diet [45,46].

### 2.2. Compositional Characteristics

As the “eighth organ of the human body”, the human gut microbiome is extremely complex and vast, and it is the most abundant space for microbial communities. Aside from its species composition, the microbiome also possesses an immense gene pool, with a total gene count approximately 150 times that of the human genome—hence, it is also referred to as the “second human genome”. The gut is responsible for absorbing 99% of nutrients, making it the body’s largest digestive organ. At the same time, 70–80% of the immune cells are gathered here, becoming the largest immune organ. Additionally, the gut contains approximately 500 million neurons that profoundly influence emotional states, earning it the nickname “the body’s second brain”.

**Figure 3:**
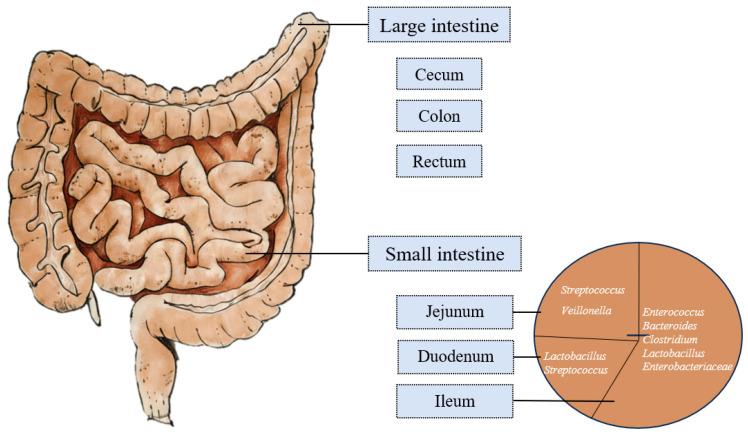
Distribution of the main flora in the small intestine.

The small intestinal microbiome serves as a transitional ecological zone connecting the stomach and large intestine, functioning as the primary site for nutrient digestion and absorption [47]. The distribution of the main flora in the small intestine is shown in Figure 3. The composition and density of the small intestinal microbiome exhibit a distinct longitudinal gradient, divided into the three following segments: ① In the duodenum, the microbial density was low (10^3^ CFU/mL), with common bacterial genera including *Lactobacillus* and *Streptococcus*. ② In the jejunum, the microbial density was moderate (10^4^ CFU/mL), with common bacterial genera including *Lactobacillus*, *Streptococcus*, and *Veillonella*. ③ In the ileum, the microbial density was increased to 10^6–^10^8^ CFU/mL, with common bacterial genera including *Enterobacteriaceae*, *Enterococcus*, *Bacteroides*, *Clostridium*, *Lactobacillus*, and *Veillonella*.

The longitudinal distribution from the cecum to rectum is divided into the three following segments: ① cecum/ascending colon: The primary site of fermentation, rich in bacteria adept at fermenting fresh dietary fiber. ② Transverse colon/descending colon/sigmoid colon: With the absorption of water and electrolytes, the contents gradually take shape and the pH approaches neutrality. The microbial community composition stabilizes, and fermentation activity decreases. ③ Rectum: The composition of the microbiome was very similar to that of fecal samples, but the mucosa-associated microbiome still maintained its uniqueness. Common bacterial genera include *Bacteroides*, *Eubacteria*, *Bifidobacterium*, *Ruminococcus*, *Peptostreptococcus*, *Lactobacillus*, *Escherichia*, and *Lactococcus*.

### 2.3. Core Functions—“Hidden Organs” from Metabolism to Immunity

The gut microbiome not only coexists with us but also plays an indispensable role in human nutritional metabolism. Its core function is to decompose substances that cannot be digested by the body itself, synthesize essential nutrients, and regulate energy balance. The number of genes contained within the gut microbiome far exceeds that of the human genome itself. These genes encode numerous enzymes not naturally present in the human body and participate in the metabolism of nutrients such as food components, amino acids, and vitamins [48]. The gut microbiome prevents harmful pathogens from colonizing and multiplying within the intestine through a “space-occupying” effect. Simultaneously, microbial metabolites contribute to immune regulation by modulating immune cell activity, orchestrating intestinal immune homeostasis and enhancing resistance to pathogens. Microbial metabolites can also regulate the activity of immune cells, maintaining intestinal immune homeostasis and resisting pathogen invasion [49]. There is a complex “gut–brain axis” interaction between gut microbes and the brain [50]. Microbes can produce various neuroactive substances, such as serotonin, dopamine, and gamma-aminobutyric acid (GABA) [51,52], which can directly or indirectly influence mood, cognition, and behavior. Studies have shown that gut microbiota dysbiosis is associated with neuropsychiatric disorders such as anxiety, depression, and Parkinson’s disease [53,54].

#### 2.3.1. Nutrition Energy Metabolism

① Carbohydrate metabolism: The primary sources of energy and nutrients available to the human gut microbiota are carbohydrates derived from both the host and dietary intake. On the one hand, the gut microbiota converts digestible carbohydrates into glucose and other forms of monosaccharides through the action of small intestinal hydrolases. On the other hand, indigestible carbohydrates are transported to the colon by fermentation with bacteria such as *Clostridium*, *Bacteroides*, and *Prevotella*. The primary products of colonic fermentation are SCFAs, including acetic acid that enters the bloodstream, participates in systemic metabolism, and serves as a precursor for cholesterol and fatty acid synthesis [55]. Propionic acid is primarily absorbed by the liver; it inhibits cholesterol synthesis and serves as a substrate for gluconeogenesis, thereby aiding in the regulation of blood glucose levels and appetite [55]. Butyric acid is the primary energy source for colonic epithelial cells. It maintains the integrity of the intestinal barrier, possesses anti-inflammatory properties, and helps prevent colorectal cancer. SCFAs not only provide energy to intestinal cells but also act as signaling molecules that affect metabolic health, immune regulation, and appetite control [56,57].
② Lipid metabolism: The gut microbiome influences host lipid metabolism through multiple mechanisms. The gut microbiome possesses the capacity to regulate dietary lipid composition, digestion, and absorption and can modify intestinal lipoprotein formation. *Lactobacillus*, *Enterococcus*, *Clostridium*, and Proteobacteria in the gut can reduce glycerol to 1,3-propylene glycol. The gut microbiome can regulate fat storage by inhibiting adenosine monophosphate kinase (AMPK) and fasting-induced adipose factor (FIAF) [58,59].
③ Protein and amino acid metabolism: The gut microbiome, on the one hand, secretes proteases that hydrolyze proteins into oligopeptides and amino acids; on the other hand, they produce various metabolic by-products through fermentation, for example, SCFAs, sulfides, aromatic compounds, polyamines, etc. Microbial protein metabolism is a double-edged sword, and its equilibrium is crucial for gut health. Protein glycolysis products involved in intestinal flora have a wide range of effects on intestinal homeostasis, the immune barrier, and inflammation within the body [60,61,62].

#### 2.3.2. Immunological Education and Homeostasis

The microbiome plays a central role in training and shaping the host’s immune system. During infancy and early childhood, the microbial colonization of mucosal tissues helps the immune system to distinguish between the “self” and “non-self”, thereby establishing immunological memory. This concerns human health and disease, preventing excessive reactions to harmless substances. The role of the gut microbiome in host immunity is primarily manifested in the development of the immune system, the regulation of immune cells, the maintenance of intestinal barrier function, and the modulation of systemic immune responses.

① Development of the immune system: The gut microbiome serves as a crucial stimulus for the development of the neonatal intestinal immune system [63,64]. Through their interaction with intestinal epithelial cells, they promote the maturation of gut-associated lymphoid tissue and induce the differentiation and balance of immune cells [65].
② Immune cell regulation—T cell differentiation and modulation: Within mesenteric lymph nodes, naive T cells differentiate into Th1, Th2, Th17, or regulatory T cells, influencing cytokine secretion to maintain local intestinal and systemic immune homeostasis [66]. For instance, filamentous bacteria can induce Th17 cell differentiation, whilst polysaccharide A produced by Bacteroides fragilis stimulates regulatory T cell (Treg) differentiation, counteracting Th17 and Th1 cell responses [67,68]. B cell antibody production: The gut microbiome can influence B cell class switching and secretion, stimulating B cells in gut-associated lymphoid tissue to produce IgA [69]. The gut microbiome and its metabolites can influence the migration and cross-reactivity of immune cells. For example, Proteobacteria, as a keystone species, can promote the migration of innate lymphoid cells (ILC2s and ILC3s) from the gut to the lungs and participate in the maintenance of airway mucosal immunity [70].
③ Maintaining intestinal barrier function: The gut microbiome interacts with intestinal epithelial cells to promote the expression of tight junction proteins, thereby reinforcing the tight junctions between epithelial cells and forming a physical barrier [71]. The gut microbiome can also produce antimicrobial substances that inhibit the colonization and proliferation of potentially harmful microorganisms, thereby safeguarding the stability of the intestinal microenvironment [72,73].
④ The role of metabolites in immune regulation: SCFAs comprise one of the primary metabolites produced by anaerobic bacteria during the fermentation of dietary fiber and resistant starch. SCFAs can regulate the function of immune cells by directly or indirectly inhibiting histone deacetylases (HDACs). For example, sodium butyrate reduces the secretion of TNF-α in human peripheral blood mononuclear cells (PBMCs) following LPS induction and inhibits NF-κB activity within PBMCs in vitro [74]. The gut microbiome can also metabolize tryptophan to produce indole and its derivatives, among other immunologically active metabolites. These metabolites regulate immune cell function by activating the aryl hydrocarbon receptor (AHR) [75].

#### 2.3.3. Neuroendocrine Regulation

The role of the gut microbiome in regulating the host nervous system is primarily manifested through bidirectional interactions with the host nervous system via the gut–brain axis, influencing neurodevelopment, emotional behavior, and the onset of neurological disorders. Specific effects are as follows:

① Bidirectional communication mechanism of the gut–brain axis: i. Microbe-derived metabolites (such as SCFAs and neurotransmitter precursors) enter the circulatory system, crossing the blood–brain barrier to influence the central nervous system [76]. ii. Immune signaling: The gut microbiome activates immune cells, regulating neuroinflammation and neurodevelopment through cytokine secretion [77]. iii. Hormonal circulation: Enteric endocrine cells release hormones (such as serotonin) in response to microbial cues, which are transported via the bloodstream to the brain to regulate mood and cognition [78]. iv. Neural pathways: Gut microbiome-induced signals, such as vagal transmission to the central nervous system, establish direct neural connections [79].
② Profound effects on neurodevelopment: i. Critical developmental window: The colonization of the gut microbiome during the perinatal period is crucial for brain immune and neural development. The gut microbiome may influence hippocampal volume, myelination, and behavior-dependent changes [80,81]. ii. Neurotransmitter synthesis: Lactobacillus and Bifidobacterium can synthesize gamma-aminobutyric acid (GABA, the brain’s primary inhibitory neurotransmitter); Candida, Escherichia coli, and Enterococcus produce serotonin (involved in mood regulation). A study revealed that another bacterial metabolite, succinate, has a protective effect on dopaminergic neurons in the substantia nigra [82]. iii. Microglial function: The gut microbiome modulates microglial maturation via dendritic cells, thereby influencing neuroinflammatory responses.
③ Regulation of emotions and behavior: i. Mood-related neurotransmitter: Approximately 95% of serotonin (5-hydroxytryptamine) is produced by chromaffin cells in the intestinal mucosa, where it participates in the regulation of mood and cognition. The gut microbiome influences serotonin synthesis through tryptophan metabolism; for instance, Bifidobacterium infantis increases plasma tryptophan levels, thereby promoting central serotonin transmission [83]. ii. Association of behavioral phenotypes: Preclinical studies indicate that gut microbiome dysbiosis is closely associated with neuropsychiatric disorders such as anxiety, depression, and autism spectrum disorder (ASD) [84]. For example, a depressed mood and anxiety are common in patients with postinfectious irritable bowel syndrome (IBS) [85].

## 3. Meta-Omics Reveals the Interaction Between the Gut Microbiome and the Host

In the 21st century, human beings entered the post-genomic era. Metagenomics, metatranscriptomics, metaproteomics, and metametabolomics are prominent in studies and have been applied to the study of the intestinal microbiome. The microbiome and the host genome do not operate independently. Studies have shown that the gut contains hundreds of bacterial species, thousands of strains, and millions of bacterial genes which are closely related to human health and disease. The host’s genetic background influences the composition of its microbiome, which in turn affects the expression and function of host genes through multiple pathways, such as metabolic products and immune regulation (Table 1).

### 3.1. Functional Analysis of Meta-Omics

Metagenomics: In 1998, Handlsman et al. proposed the concept of metagenomics, aiming to study the collective DNA of all microorganisms within an environment. It provides a “genetic catalogue” of the microbiome. Not only does it comprehensively reveal microbial communities, but it also enables the prediction of the functions of all genes within the microbiome [86,87].

Metatranscriptomics: In 1997, Dr. Velculescu proposed the concept of the transcriptome for the first time. This is the sum of all mRNAs expressed by a given cell at a given time. This approach reveals the gene expression patterns and transcriptional regulatory mechanisms within microbial communities, thereby reflecting the dynamic changes in microbial gene expression under specific environmental conditions. Researchers must investigate the gene expression and function of human microbial communities, such as the gut microbiome, to understand their role in health and disease [88,89].

Metaproteomics: In 2006, Wilmes et al [90] redefined the concept of metaproteomics, defined as the sum total of all proteins produced by an environmental microbial community over a specific period of time. It reveals the composition and abundance of proteins, protein modifications, and protein interactions by directly extracting all microbial proteins from environmental samples, followed by high-throughput protein identification and quantitative analysis, thereby elucidating the phenotypic and functional state of microbial communities [91,92]. This serves to reveal the interaction mechanism between pathogens and hosts and provide new ideas for disease diagnosis and treatment.

Macrometabolomics: The concept of metabolomics proposed by Professor Jeremy Nicholson in 1999 laid the theoretical foundation for the development of macrometabolomics [93]. It reveals the metabolic activities and regulatory mechanisms of microbial communities [94]. Studying the metabolites and metabolic pathways of human microbial communities (such as intestinal microbiome), discovering microbiome-derived active molecules, diagnosing disease biomarkers, and studying intestinal microbiome–host co-metabolism have high clinical translational value [95,96].

### 3.2. Microbiome–Host Gene Interactions

Regulation of the microbiome on the host genome: ① Epigenetic modification: Intestinal microbes affect host epigenetic modification through metabolites such as SCFAs. For example, as histone deacetylase inhibitors (HDACIs), SCFAs can regulate DNA methylation and the histone acetylation levels of host genes, thereby affecting gene expression [97]. Propionate derived from the gut microbiome induces specific DNA methylation, thereby predisposing obese individuals to diabetes [98].

② Transcription factor binding: Microbiome metabolites may directly bind to host transcription factors, thereby regulating gene expression. For instance, indole and its derivatives act as ligands for the AHR, modulating host inflammatory and immune responses by activating AHR [75].

③ Chromatin remodeling: The microbiome influences gene transcription by modulating host chromatin accessibility. For instance, ATAC-seq analysis of co-cultured colonic epithelial cells with microbes revealed that specific microorganisms alter chromatin openness in host tissues, thereby regulating transcription factor binding and gene expression.

④ Alternative splicing regulation: The microbiome participates in disease pathogenesis by influencing alternative splicing events in host genes, for instance, in the application of splicing-related methods in the clinical diagnosis and treatment of IBD [99].

Host genome regulation of the microbiome: ① Immune pathway regulation: Host genes directly regulate the microbiome’s structure through immune signaling pathways (such as TLR signaling). The gut microbiome can activate downstream signaling cascades by engaging specific TLRs, thereby modulating metabolic and inflammatory gene expression. Among these, TLR2 tunes host defense by controlling goblet cell terminal differentiation and mucin production [100,101]. ② Metabolic pathway regulation: Host genes influence microbiome composition via metabolic pathways such as bile acid synthesis. ③ Genetic variation correlation: Significant associations exist between host genetic variation and microbiome characteristics.

### 3.3. Bidirectional Regulation Is Associated with Diseases

Gut microbes bidirectionally regulate the host immune system through metabolites (such asSCFAs, bile acids) and cytokines (such as IL-22). The gut microbiome has coexisted and coevolved with humans over long periods of time, playing a vital role in the body’s metabolic activities, particularly in fat metabolism. Therefore, gut microbiome imbalance is associated with the onset and progression of numerous diseases related to lipid metabolism disorders, such as hyperlipidemia, fatty liver disease, obesity, and diabetes. In addition to genetic and environmental factors believed to be associated with the development of autoimmune diseases, alterations in the composition of the gut microbiome are also considered one of the reasons for the rapid increase in autoimmune diseases. An increasing number of studies has shown that changes in the intestinal microecology are closely related to metabolic and immune diseases. Therefore, altering dietary or gut microbiome composition to increase microbial abundance and metabolite production may serve as a novel therapeutic strategy for these diseases.

**Table 1:** Bacteria and metabolites related to common metabolic and immune system diseases.

Items	Diseases	Microbiome	Metabolites	Reference
Metabolic System	Obesity	*Christensenellaceae*⬇	--	[102]
		*g_Oscillibacter*⬆	Caproate⬆	[103]
		*c_Betaproteobacteria, f_Sutterellaceae, g_Enterobacter, and s_Bacteroides_vulgatus*⬇	Isobutyrate⬇	[103]
	T2DM	*Desulfovibrio piger*, *Peptostreptococcus*	--	[104]
		--	IPA⬇	[105]
	hyperlipidemia	*Lactobacillus gasseri RW2014*⬇	bile acids⬇	[106]
	CVD	*Roseburia intestinalis*⬇	--	[107]
		--	TMAO⬆	[108]
	MASLD	*Bifidobacterium bifidum*⬇	IAA⬇	[109]
Immune System	MS	*Eubacterium hallii, Butyricoccaceae, Blautia*⬇	SCFA⬇	[110]
	RA	*Escherichia*	Ricinoleic acid, Xanthurenic acid, Quinoline-2,8-diol ⬇	[111]
	T1D	Butyrate-producing bacteria⬇	Butyrate⬇	[112]

The upper arrow indicates an increase in content, and the lower arrow indicates a decrease in content.

## 4. Application

### 4.1. In Vitro Model

Single-bacterium CRISPR editing utilizes the CRISPR-Cas system to perform gene editing on individual strains within the gut microbiome, enabling the precise knockout or insertion of specific genes. Thus, we investigate the roles of these genes in microbial metabolism, pathogenicity, and host interactions. An organoid–microbe co-culture involves co-culturing intestinal organoids with the gut microbiome to simulate the intestinal microenvironment, thereby studying the relationship between microbes and disease. Additionally, in vitro model technologies such as the gut-on-a-chip provide powerful tools for studying the interactions between the gut microbiome and their hosts, aiding in achieving a deeper understanding of the mechanisms by which microbes influence health and disease [113,114].

### 4.2. In Vivo Model

Implanting single strains (such as specific probiotics) or composite strains (Schaedler microbiome) into germ-free mice or performing fecal transplants serves as a powerful approach for studying the effects of specific microorganisms. Through xenografting, the microbiome from the human host’s gut is transplanted into germ-free mice, enabling the transfer of disease phenotypes to these mice. This establishes corresponding animal models for studying the corresponding human diseases [115]. In recent years, humanized mouse models have been extensively applied in various research areas, including studies on intestinal diseases, antibiotic treatments, fecal transplants, gluten-induced immunopathological features, and obesity. They are also utilized to investigate interactions between the gut and the microbiome, as well as gene expression profiles or metabolomic characteristics within the intestine.

### 4.3. Advanced Computing Technology

The development of machine learning is inextricably linked to the advancement of artificial intelligence [116]. It is widely accepted that machine learning originated in the mid-1950s [117].

Since the beginning of the 21st century, with the increase in data volume and computational power, deep learning has become a hot topic in machine learning [118,119]. For example, deep learning models can predict from metagenomic data which microbial communities are sensitive to specific drug responses [120,121]. The high dimensionality, complexity, nonlinearity, and over-dispersed nature of gut microbiome data determine the applicability of machine learning in gut microbiome research [122]. For instance, many studies have found that by clustering the composition of the intestinal microbiome of volunteers, different intestinal shapes can be constructed, and the relationship between diet and metabolic health presents different results in different intestinal shapes [123]. Given that the gut microbiome influences individual variations in dietary metabolism, machine learning algorithms can integrate gut microbiome and host phenotype data to predict personalized dietary responses [124]. It also enables multi-omics data integration, such as MWAS and causal machine learning, which utilize machine learning algorithms to infer causal relationships between microbial communities and host phenotypes [125,126].

## 5. Gut Microbiome and Precision Medicine

At present, a primary mechanism underlying the therapeutic effects of gut microbiome-modulating drugs is the biotransformation of these drugs by the microbiome. A series of metabolic transformations, including reduction reactions, hydrolysis reactions, and functional group transfer, are mediated through the action of gut microbial enzymes [127]. For example, Xu’s group analyzed by high-resolution mass spectrometry and targeted metabolomics that Plantamajoside was metabolized into the following active metabolites in gut microbes: calceolarioside A, dopaol glucoside, hydroxytyrosol, caffeic acid, and 3-HPP. Similarly, the active compounds in Plantamajoside also influence the gut microbiome’s metabolism of SCFAs and tryptophan [128]. A healthy microbiome is usually characterized by high species diversity and stability. Studies have found that many chronic diseases, such as inflammatory bowel disease, obesity, type 2 diabetes, and other diseases, are associated with dysbiosis of the microbiome. This condition manifests as a reduction in beneficial bacteria, an increase in harmful bacteria, or a decrease in species diversity. Therefore, the composition of the microbiome can serve as a potential biomarker for assessing health status and disease risk. By modulating the gut microbiome, the efficacy of drugs can be significantly improved and side effects reduced. The U.S. Food and Drug Administration approved fecal microbiome transplantation (FMT) as an investigational new drug in 2013. Research indicates that FMT alleviates ulcerative colitis (UC) and irritable bowel syndrome (IBS) by restoring intestinal ecological balance [129,130].

Additionally, significant progress has been made in the application of engineered bacteria. For example, applying the probiotic *Escherichia coli* Nissle 1917 as the host in model animals revealed its impact on *Pseudomonas aeruginosa* within the body [131]. These findings support the further development of engineered microorganisms with active properties for preventing and treating intestinal infections. Yang’s group demonstrates a novel strategy that enhances immune responses against tumors and the efficacy of CAR-NK cell therapy by engineering bacterial surfaces to display immune-activating molecules, offering new possibilities for cancer treatment [132]. Researchers have also developed a “GlycoCaging” strategy that leverages the metabolic activity of specific gut bacteria to precisely deliver drugs to target areas, enhancing therapeutic efficacy while minimizing side effects.

In summary, we comprehensively reviewed the composition and function of the gut microbiome, combined with modern high-throughput sequencing technology and multi-omics research methods. Future studies in the field will continue to underline the interactions between microbes and their hosts, mechanisms of action of the microbiome in the occurrence and development of diseases, as well as potential new therapeutic strategies based on microbes and their products.

## Data Availability

No new data were created or analyzed in this study.

## Abbreviations

ATAC-Seq	Assay for transposase-accessible chromatin by sequencing
CAR-NK	Chimeric antigen receptor natural killer cell
CFU	Colony-forming unit
CRISPR	Clustered regularly interspaced short palindromic repeats
CVD	Cardiovascular disease
IAA	Indole-3-acetic acid
IgA	Immunoglobulin A
IL-22	Interleukin-22
ILC2s	Group 2 innate lymphoid cells
ILC3s	Group 3 innate lymphoid cells
IPA	Indole-3-propionic acid
LPS	Lipopolysaccharide
MASLD	Metabolic dysfunction-associated steatotic liver disease
MS	Multiple sclerosis
MWAS	Metabolome-wide association study
NF-κB	Nuclear factor-kappa B
RA	Rheumatoid arthritis
SCFAs	Short-chain fatty acids
T1D	Type 1 diabetes
TH1	T helper cell 1
TH2	T helper cell 2
TH17	T helper cell 17
TLR	Toll-like receptor
TMAO	Trimethylamine N-oxide
TNF-α	Tumor necrosis factor-alpha
UDCA	Ursodeoxycholic acid
3-HPP	3-(3-hydroxyphenyl) propionic acid
